# Comparative attainment of 5-year undergraduate and 4-year graduate entry medical students moving into foundation training

**DOI:** 10.1186/1472-6920-9-76

**Published:** 2009-12-22

**Authors:** Gillian Manning, Paul Garrud

**Affiliations:** 1 School of Graduate Entry Medicine and Health, University of Nottingham, Medical School, Royal Derby Hospital, Uttoxeter Road, Derby DE22 3DT, UK

## Abstract

**Background:**

Graduate entry medicine is a recent innovation in UK medical training. Evidence is sparse at present as to progress and attainment on these programmes. Shared clinical rotations, between an established 5-year and a new graduate entry course, provide the opportunity to compare achievement on clinical assessments. To compare completion and attainment on clinical phase assessments between students on a 4-year graduate entry course and an established 5-year undergraduate medicine course.

**Methods:**

Overall completion rates for the 4 and 5 year courses, fails at first attempt, and scores on 14 clinical assessments, were compared between 171 graduate-entry and 450 undergraduate medical students at the University of Nottingham, comprising two graduating cohorts. Percentage assessment marks were converted to z-scores separately for each graduating year and the normalised marks then combined into a single dataset. Z-score transformed percentage marks were analysed by multivariate analysis of variance and univariate analyses of variance for each summative assessment. Numbers of fails at first attempt were analysed aggregated across all assessments initially, then separately for each assessment using χ^2^.

**Results:**

Completion rates were around 90% overall and significantly higher in the graduate entry course. Failures of assessments overall were similar, but a higher proportion of graduate entry students failed the final OSLER. Mean performance on clinical assessments showed a significant overall difference, made up of lower performance on 4 of 5 knowledge-based exams (as well as higher performance on the first exam) by the graduate entry group, but similar levels of performance on all the skills-based and attitudinal assessments.

**Conclusions:**

High completion rates are encouraging. The lower performance in some knowledge-based exams may reflect lower prior educational attainment, a substantially different demographic profile (age, gender), or an artefact of the first 2 years of a new graduate entry programme.

## Background

In 1997, the UK Medical Workforce Standing Advisory Committee (MWSAC) recommended that clinical courses with graduate entry should be developed. The underpinning rationale was to increase the size and diversity of medical student intake and allow the faster production of doctors to address a predicted workforce shortage [1]. In addition UK universities were encouraged to widen access to higher education, particularly in terms of socioeconomic disadvantage. Graduate students are known to bring a range and wealth of undergraduate and life experiences to learning [2]. Some graduate entry programmes are claimed to minimise the effects of disadvantage, increasing the representation of students from diverse backgrounds, achieving a better match between the medical student population and the general population, and encouraging more flexible and inclusive selection and admissions policies [3]. Graduate entry students have usually taken a mature decision to study medicine, thus improving retention rates of junior doctors [3,4].

This shift to graduate entry has been accompanied by a move to a more learner-centred and problem oriented approach (GMC, 1993, 2002). Some have argued that these changes have reduced student knowledge of the basic and clinical sciences and that this shift in learning is compromising learning in the clinical phases of a medical degree [5].

The University of Nottingham (UoN), in partnership with Derby Hospitals NHS Foundation Trust, launched a 4-year graduate entry medicine (GEM) course in 2003 admitting graduates with a minimum of a 2:2 degree in any subject. The course seeks to encompass the requirements of 'Tomorrow's Doctors' (2002) through problem-based learning with a strong emphasis on personal and professional development and early clinical experience. Over the 18-month, mainly pre-clinical, phase of the course students study the basic and clinical sciences, begin to learn and develop clinical skills and professionalism. In addition, the university continues to run a 5-year undergraduate course (UG). The structure of the UG pre-clinical course is integrated and systems-based in the first two years. This is followed by a 6-month period with the emphasis on research, including a research project and dissertation, that, together with the work of the first clinical phase, leads to the award of a BMedSci. Students following the 4-year graduate and 5-year undergraduate entry then merge to a single cohort to undertake 2.5 years of full-time clinical rotations.

Debate around the early and long-term outcomes of students from graduate entry medicine programmes thrives, and with the increasing number of graduating entry students entering postgraduate training in the UK this will continue.

The structure of the medical degree in Nottingham, with separate intakes of undergraduate and graduate entry students, who then merge and follow the same clinical training provides an opportunity to measure the attainment of both sets of students. Therefore, the aim of this study was to compare the absolute and relative attainment of students graduating from our medical school in 2007 and 2008 during and at the end of 2.5 years of clinical training to determine similarities and differences between the UG and GEM groups, and to investigate factors affecting attainment.

## Methods

Clinical training spans 2.5 years with regular (n = 14) summative assessment of knowledge and skills and at the end of each clinical attachment student attendance and professional behaviour is assessed by the clinical supervisors and students not meeting the defined criteria are barred from sitting summative assessments. An overview of the clinical training programme is shown in Table 1. With the approval of the University of Nottingham medical school ethics committee, assessment data from the clinical summative assessments, for all students graduating with the Nottingham medical degree in 2007 (n = 320) and 2008 (n = 325) was collected.

**Table 1:** Overview of Clinical Training

Clinical Phase 1 17 weeks	Clinical Phase 2 40 weeks	Clinical Phase 3 36 weeks
Introduction to Medicine & Surgery	Child Health, Obstetrics & Gynaecology, Health Care for the Elderly (HCE), Psychiatry, Specials (Ear Nose Throat, Dermatology, Ophthalmology	Advanced Clinical Experience (ACE)

	**Knowledge-based summative examinations**	

MCQ Clinical Case Based questions integrating basic science and clinical knowledge	MCQ in all specialities Clinical Case Based questions integrating basic science and clinical knowledge	MCQ

	**Skills-based summative examinations**	

OSLER	OSCEs in Psychiatry, Obstetrics & Gynaecology; OSLER in Child health	OSCE + OSLER

### Data Analysis

The percentage marks and numbers of fails for each of the 14 summative assessments were collated. Scores for any student with an interruption to their course (i.e. who did not graduate after the usual 4 or 5 year period since entry) were discarded. Only assessment marks from the first attempt were analysed, discarding reassessment marks for students who failed at first attempt. Analyses were conducted using χ^2^.

In order to eliminate variation between the two graduating cohorts (2007, 2008), percentage assessment marks were then converted to z-scores separately for each graduating year and the normalised marks then combined into a single dataset. Z-score transformed percentage marks were analysed by multivariate analysis of variance for initial comparison of GEM and UG attainment, followed by univariate analyses of variance for each summative assessment. Numbers of fails at first attempt were analysed aggregated across all assessments initially, then separately for each assessment using χ^2^.

## Results

From the initial pool of 645 graduates in 2007 and 2008, exclusion of those who had taken longer than the standard 4 (or 5) years left 306 graduates from 2007 and 315 from 2008, 171 who completed the GEM course, and 450 who completed the UG course. Median ages at entry were 18 and 28 years and the proportion of women were 63% and 46% for UG and GEM groups respectively.

The proportions of initial entrants to the two courses that graduated after uninterrupted progress were high: GEM 94% (171 from 182 entrants); UG 90% (450 from 500 entrants), this difference being statistically significant (p = 0.04).

Multivariate analysis of variance showed overall that the GEM students performed significantly differently from the UG students (F = 5.5, df 14&599, p < 0.001); year of graduation had no significant effect (p = 0.60), but there was a significant interaction between graduation year and GEM vs. UG (F = 4.2, df 14&599, p < 0.001), comprised of the clinical phase 3 OSCE and OSLER (see below). Univariate analyses of variance showed that this comprised a mixture of differences as summarised in Table 2. This shows that the GEM graduates performed significantly better on their first clinical knowledge exam than the UG group, but significantly worse on 4 out of the 5 subsequent knowledge-based exams. There were no significant differences between the GEM and UG groups overall on any skills-based assessment (OSCE or OSLER); but significant interactions (all p values < 0.001) with graduation year for the two skills-based assessments in the final clinical phase (GEM worse than UG in 2007, GEM better than UG in 2008). The GEM group performed better than the UG group on one other assessment - a learning journal used in the healthcare of the elderly attachment.

**Table 2:** Comparison of mean z-score attainment

Clinical phase	Assessment	GEM	UG	p
1	MCQ	0.17	-0.07	0.01**
	
	OSLER	0.10	-0.03	0.14

2	Child health MCQ	-0.15	0.06	0.02*
	
	Child health OSLER	0.10	-0.03	0.16
	
	O&G MCQ	-0.10	0.03	0.13
	
	O&G OSCE	0.03	-0.01	0.62
	
	Psychiatry MCQ	-0.14	0.05	0.03*
	
	Psychiatry OSCE	-0.01	0.00	0.92
	
	HCE learning journal	0.17	-0.05	0.01**
	
	Specials MCQ	-0.19	0.07	0.00**

3	ACE MCQ	-0.19	0.08	0.00**
	
	ACE OSCE	-0.05	0.02	0.48
	
	ACE OSLER	-0.07	0.03	0.25

* p < 0.05 ** p < 0.01

The number failing each assessment at first attempt is summarised in Table 3. Summing all failures over the thirteen assessments, there was no significant difference overall in numbers of failures (p = 0.26). Examining failures in each individual assessment revealed that there were no significant differences in 12 of the 13, but that there was a higher proportion of failures amongst the GEM group than the UG group on the CP3 OSLER (p = 0.04). Most students who failed assessments failed only one: there was no significant difference between the GEM and UG group in the numbers failing multiple assessments (p = 0.21).

**Table 3:** Comparison of failures at first attempt

Clinical phase	Assessment	GEM Numbers (%)	UG Numbers (%)	p
1	MCQ	7 (4.1%)	27 (6.0%)	0.35
	
	OSLER	3 (1.8%)	11 (2.4%)	0.60

2	Child health MCQ	3 (1.8%)	9 (2.0%)	0.84
	
	Child health OSLER	2 (1.2%)	4 (0.9%)	0.75
	
	O&G MCQ	2 (1.2%)	3 (0.7%)	0.54
	
	O&G OSCE	0 (0%)	1 (0.2%)	0.54
	
	Psychiatry MCQ	0 (0%)	0 (0%)	1.00
	
	Psychiatry OSCE	7 (4.1%)	12 (2.7%)	0.36
	
	HCE learning journal	0 (0%)	1 (0.2%)	0.54
	
	Specials MCQ	2 (1.2%)	2 (0.4%)	0.32

3	ACE MCQ	0 (0%)	0 (0%)	1.00
	
	ACE OSCE	14 (8.2%)	31 (6.9%)	0.59
	
	ACE OSLER	5 (2.9%)	4 (0.9%)	0.04*

* p < 0.05 ** p < 0.01

## Discussion

Overall, the two streams of students - UG and GEM - were very similar in terms of their competence: similar, high proportions completing their medical degree within the standard period. This finding fits well with the national picture in the UK where over 90% of medical students complete successfully [6] and internationally (80.6-82.2% in 4 years, 91.3% in 5 years in US:[7]); higher proportions of entrants completing university courses in medicine than over all subjects in the UK (78.1% for first degrees in 2004-5: [8]).

Examination of the relative performance of GEM and UG students on clinical assessments showed broadly similar proportions of first attempt fails and mean levels of attainment. However, there was a number of individual knowledge-based assessments where the two groups differed significantly. The pattern is that of two groups diverging over time, as they progress through the shared clinical phases. Thus GEM are better than UG in clinical phase 1, then deteriorate and are worse than UG through most of clinical phase 2 and then clinical phase 3, though with similar proportions of fails. A possibility is that this difference shows up as an artefact of the lower drop out rate amongst GEM compared to UG students, but we have no evidence on this point.

It is pertinent to consider what these results tell us about how the graduate entry students may differ from the undergraduates. The first possibility is that poorer performance, especially in a number of knowledge-based assessments represents a genuine trend that reflects weaker prior educational attainment. The data available (UCAS tariff score; class of first degree) are consistent with this suggestion, though tariffs are only available for about half the GEM entrants, and first degree class for the UG students comprises the bachelor of medical sciences degree that is awarded after 3 years on the 5-year medicine course. That the somewhat poorer performance can be seen in knowledge-based assessments, typical of prior educational experience, but not in skill-based assessments, also is suggestive. A different pattern of results has recently been reported by [9] where graduate entry students outperformed those following a 5-year medicine course on knowledge-based assessments: however, it seems likely that this is related to differences in student profile with a higher requirement for first degree class (minimum 2.1 vs. 2.2), and a younger median age (24 vs 28 years) in that programme.

The second possibility is that differences in performance are related to the different demographic profile of the GEM and UG streams, the most prominent being the higher age of the GEM cohorts and the lower proportion of women. Secondary educational attainment in the UK has been greater amongst women than men for several decades [10], though it is probably unwise to generalise to the highly self-selected group who apply to medicine. Conclusions about the influence of demography on attainment of GEM students must also await evidence that the profile of entrants has stabilised since average age has been steadily declining since the introduction of graduate entry in the UK in 2001 [11]. It is also sensible to register that the 2007 graduates included the first ever cohort of GEM students at Nottingham: the significant cohort by GEM vs UG effect seen in the final year OSCE and OSLER may indicate a particular difficulty for that first GEM cohort. Lastly, any individual assessment may be prone to error in measuring attainment, and examination of a larger data set a few years hence should demonstrate whether a consistent pattern is in evidence.

The study strength is the direct comparison of performance during the same full-time clinical rotations for both GEM and UG groups. Weaknesses are the relatively small sample sizes (171, 450) that limit the statistical power, and the difficulty in generalising these results to different graduate entry medicine curricula.

## Conclusions

Whilst completion rates were high in both groups the higher completion rate for GEM students is encouraging. The lower performance of GEM students in some knowledge-based exams may reflect lower prior educational attainment, a substantially different demographic profile (age, gender), or an artefact of the first 2 years of a new graduate entry programme.

## List of Abbreviations

ACE: Advanced Clinical Experience; GEM: Graduate Entry Medicine; HCE: Health Care for the Elderly; MCQ: Multiple Choice Question; OSCE: Objective Structured Clinical Examination; OSLER: Objective Structured Long Examination Record; UG: Undergraduate.

## Competing interests

The authors declare that they have no competing interests.

## Authors' contributions

PG jointly conceived the study and methods, cleaned and analysed the data, and jointly wrote the method, results and discussion. He is the Guarantor. GM jointly conceived the study and methods, wrote the introduction and jointly wrote the method, results and discussion. Both authors read and approved the final manuscript.

## Pre-publication history

The pre-publication history for this paper can be accessed here:

http://www.biomedcentral.com/1472-6920/9/76/prepub
